# Rhein: An Updated Review Concerning Its Biological Activity, Pharmacokinetics, Structure Optimization, and Future Pharmaceutical Applications

**DOI:** 10.3390/ph17121665

**Published:** 2024-12-10

**Authors:** Yuqi Fu, Le Yang, Lei Liu, Ling Kong, Hui Sun, Ye Sun, Fengting Yin, Guangli Yan, Xijun Wang

**Affiliations:** 1 State Key Laboratory of Integration and Innovation of Classic Formula and Modern Chinese Medicine, National Chinmedomics Research Center, National TCM Key Laboratory of Serum Pharmacochemistry, Metabolomics Laboratory, Department of Pharmaceutical Analysis, Heilongjiang University of Chinese Medicine, Heping Road 24, Harbin 150040, China; fuyq.hljcm@hotmail.com (Y.F.); liulei7711@126.com (L.L.); 15244624557@163.com (L.K.); 17748334626@163.com (F.Y.); gancaosuan@163.com (G.Y.); 2 State Key Laboratory of Dampness Syndrome, The Second Affiliated Hospital Guangzhou University of Chinese Medicine, Dade Road 111, Guangzhou 510006, China; leyang92gzy@sina.com (L.Y.); sunye0126@126.com (Y.S.)

**Keywords:** rhein, TGF-*β*1, NF-*κ*B, pharmacokinetics, twin drug, derivatives, combination therapy

## Abstract

Rhein is a natural active ingredient in traditional Chinese medicine that has attracted much attention due to its wide range of pharmacological activities. However, its clinical application is limited by low water solubility, poor oral absorption, and potential toxicity to the liver and kidneys. Recently, advanced extraction and synthesis techniques have made it possible to develop derivatives of rhein, which have better pharmacological properties and lower toxicity. This article comprehensively summarizes the biological activity and action mechanism of rhein. Notably, we found that TGF-*β*1 is the target of rhein improving tissue fibrosis, while NF-*κ*B is the main target of its anti-inflammatory effect. Additionally, we reviewed the current research status of the pharmacokinetics, toxicology, structural optimization, and potential drug applications of rhein and found that the coupling and combination therapy of rhein and other active ingredients exhibit a synergistic effect, significantly enhancing therapeutic efficacy. Finally, we emphasize the necessity of further studying rhein’s pharmacological mechanisms, toxicology, and development of analogs, aiming to lay the foundation for its widespread clinical application as a natural product and elucidate its prospects in modern medicine.

## 1. Introduction

Rhein, also known as 4,5-dihydroxyanthraquinone 2-carboxylic acid, is a lipophilic anthraquinone compound with a long history of use in China [1]. Modern pharmacological research has shown that rhein exhibits a variety of biological activities, including hypoglycemic, lipid-lowering [2,3], anti-cancer [4,5,6], anti-inflammatory [7,8], anti-fibrotic [9,10], cardiocerebral protection [11,12], antibacterial [13,14], and antiviral [15] effects. For instance, it can resist lung cancer and breast cancer via regulating the PI3K/Akt/ERK pathway, lower blood lipids by regulating PPAR*γ*, AMPK, and Sirt1, reduce inflammation via interfering with NF-*κ*B signaling pathways, and improve organ fibrosis by acting on TGF-*β*1 and *α*-SMA. Obviously, rhein has played a significant role in the treatment of diseases affecting the liver, kidneys, digestive system, and cardiovascular system [16,17]. However, the clinical application of rhein is limited by its low water solubility, low bioavailability, and potential toxicity to the liver and kidneys. Additionally, the half-life of rhein after oral administration is very short, which makes it difficult to determine the optimal treatment time window, and all of these factors have significantly hindered its further development and clinical use [18,19]. As a result, understanding its potential pharmacological mechanisms, exploring dose–response relationships, and reducing its toxicity are crucial for promoting the clinical application of rhein.

Recently, advancements in extraction, separation, and synthesis technology have greatly supported research into the structural modification of rhein, leading to the development of numerous derivatives [20]. Studies have confirmed that most derivatives of rhein exhibit greater pharmacological activity, milder toxicity, and stronger clinical practicality compared to rhein [21,22,23]. Additionally, there are also a lot of practical applications of rhein combination therapy; by combining it with another active molecule with similar pharmacological effects, it can achieve more than twice the effect of using a single drug.

In light of these developments, this article reviews and summarizes recent domestic and international research on the pharmacological effects, mechanisms of action, pharmacokinetics, toxicology, structural optimization, and combination therapy of rhein, coving studies from January 2018 to November 2014. We aim to provide a foundation for the further development of rhein and offer guidance for its safe and effective clinical application.

## 2. Herbal Sources of Rhein and Its Development History

**Figure 1:**
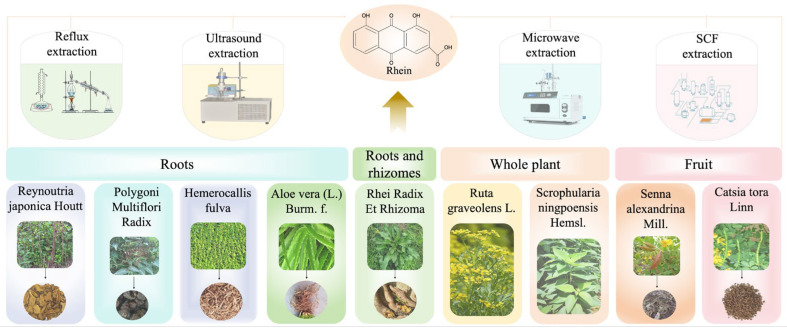
Herbal resources of rhein.

Rhein is derived from a wide range of Chinese herbal sources (Figure 1) and is mainly distributed in the roots and rhizomes of *Rheum palmatum* L., *Rheum tanguticum Maxim.ex Balf.*, or *Rheum officinale Baill.*, the roots of *Polygonum multiforum Thunb.*, *Polygonum cuspidatum Sieb.et Zucc.*, *Hemerocallis fulva.*, and *Aloe barbadensis Miller.*, the pods of *Cassia angustifolia Vahl.* and *Cassia acutifolia Delilel.*, the seeds of the *Cassia obtusifolia* L. and *Cassia tora* L., and the entire plant of *Scrophularia ningpoensis Hemsl.* and *Ruta graveolens* L. [24,25]. Rhein has been used as a medicinal substance in China for over 1000 years and is one of the main active ingredients in the well-known traditional Chinese medicine (TCM) rhubarb. Due to its extensive clinical application, the discovery and extraction of rhein monomers have become important areas of research.

The structure of rhein is shown in Figure 1, with a molecular formula of C_15_H_8_O_6_ and a molecular weight of 284.21 g/mol. It appears as coffee-colored needle-like crystals at room temperature and sublimates to obtain yellow needle crystals. Its melting point is 321–322 °C. Rhein is soluble in alkaline substances, including pyridine and dimethyl sulfoxide (DMSO), slightly soluble in alcohols, ethers, benzene, chloroform, petroleum ether, and insoluble in water [26]. Research on the synthesis, characterization, and pharmacological effects of rhein and its derivatives is often conducted with DMSO or mixed with other liquids as solvent systems [27,28].

According to comprehensive literature research, several methods have been developed for the extraction of rhein, including the reflux extraction method [29], ultrasonic extraction method [30], microwave extraction method [31], and supercritical extraction method (SCFE) [32]. Reflux extraction is the most commonly used method for extracting active ingredients from TCM, especially for compounds that are not easily decomposed by heat. Common solvents for the reflux extraction of rhein include ethanol [33], ammonia water [34], and chloroform [35]. Ultrasonic extraction technology is well-known for its high extraction efficiency, short extraction time, and low solvent consumption. It has been widely used in various fields, such as medicine, chemistry, and food, especially for extracting active ingredients from medicinal plants. Compared to traditional decoction methods, ultrasonic extraction increases the yield of rhein by threefold and enhances extraction efficiency [36].

The microwave extraction method selectively heats different components based on their ability to absorb microwave energy, allowing the separation of desired substances from the matrix [37]. Research shows that the microwave-assisted extraction of anthraquinone compounds, including rhein, provides higher yields and extraction rates. Additionally, it is more solvent-free and environmentally friendly compared to soxhlet and ultrasound extraction, making it a promising method for rapidly and efficiently extracting rhein [38]. SCFE uses a supercritical fluid to dissolve and carry specific components from the extracted material, separating them from other components. SCFE has been widely applied in the pharmaceutical field, and its rhein extraction rates are significantly higher than those of other methods [39].

While these extraction methods allow for the efficient separation of anthraquinone compounds from TCM, challenges such as noise generation and high equipment costs have limited their widespread industrial application. In addition to extraction, the chemical synthesis [40] and biotechnology [41] production of rhein have also developed significantly. For instance, chrysophanol can undergo acetylation, oxidization with chromium trioxide, and finally deacetylation under alkaline conditions to produce rhein. Another method involves dibromo ester, which undergoes hydroxyl substitution, esterification, rearrangement, hydroxyl protection, ester hydrolysis, and finally cyclization to produce rhein [42]. Napoli et al. developed a synthesis method for rhein from methoxyxylene through a series of reactions, including oxidation, esterification, Friedel–Crafts acylation, hydrolysis, sulfuric acid cyclization, diazotization, and deprotection reactions [43]. In terms of biotechnological production, rhein can be derived from emodin. Using emodin as an inducing agent, researchers isolated a strain of *Penicillium* that is tolerant to emodin. This strain can convert emodin in the culture medium to rhein with a conversion rate of 11.7%. Additionally, diacetyl rhein can be dissolved in Na_2_CO_3_ solution under dark conditions, and the extracted solution can be heated and boiled with chloroform, cooled to room temperature, and added to hydrochloric acid for acidification [44]. Subsequently, exhaust, boiling, cooling, filtration, and water scrubbing are carried out sequentially to obtain rhein crystals. Through Fries rearrangement and the decarbonization strategy, followed by cyclization in molten salt starting from dibromo ester, a synthesis of rhein was also achieved [42].

## 3. Pharmacological Activity and Clinical Application

### 3.1. Hypoglycemic and Lipid-Lowering Effects

**Figure 2:**
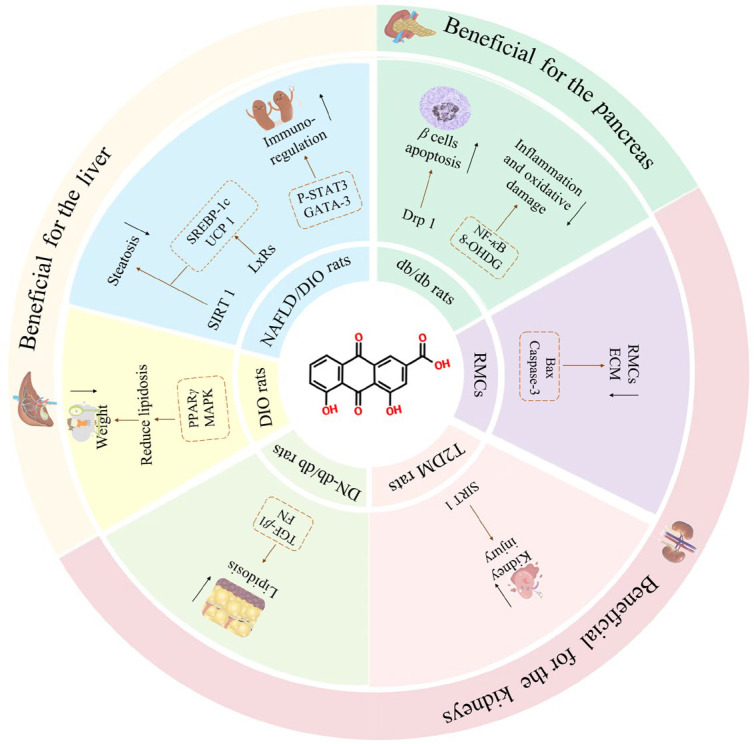
Hypoglycemic and lipid-lowering effects of rhein. The black arrow indicates the upward or downward adjustment, while the brown arrow indicates the causal relationship. NAFLD rats: Non-alcohol fatty liver disease; db/db and T2DM rats: Type 2 diabetes mellitus model; RMCs: Rat mesangial cells; DN-db/db rats: Diabetic nephropathy-type 2 diabetes model; DIO rats: Diet-induced obesity model.

Rhein regulates body glucose and lipid metabolism by inhibiting cholesterol absorption, reducing lipoprotein synthesis and ingestion, decreasing lipid accumulation, and improving insulin resistance. These effects contribute to therapeutic benefits for organs such as the liver, kidneys, and pancreas. The mechanisms of action are complex and interrelated (Figure 2).

Drp1 is a key protein in the dynein family that regulates mitochondrion division. Under high-glucose conditions, rhein can inhibit Drp1 in the mitochondrion, thereby protecting the mitochondrial ultrastructure of pancreatic B cells and reducing their apoptosis [45]. Sirt1 is another important regulator involved in processes like cell proliferation, differentiation, apoptosis, and energy metabolism in cells. It plays a crucial role in intracellular lipid metabolism, improves tissue insulin sensitivity, and prevents hepatic steatosis [46]. Studies have shown that rhein can upregulate Sirt1, significantly improving insulin resistance, dyslipidemia, and the pathological morphology of renal tissue of type 2 diabetes model rats induced by a high-glucose and high-fat diet combined with streptozotocin [47].

Non-alcoholic fatty liver disease (NAFLD) is a clinical syndrome characterized by fat accumulation and degeneration in liver cells. Previous studies have found that Sirt1 expression levels are lower in NAFLD patients compared to healthy individuals. Sirt1 also regulates adenosine 5’-monophosphate-activated protein kinase (AMPK) phosphorylation [48,49], which has been shown to reduce lipid production, enhance fatty acid oxidation, and ultimately inhibit the development of NAFLD when it is activated [50]. In a study using a NAFLD mouse model and an AML-12 cell line, rhein treatment was shown to decrease triglyceride (TG) levels in AML-12 cells and reduce serum levels of TG, total cholesterol (TC), malondialdehyde (MDA), alanine transaminase (ALT), aspartate transaminase (AST), and non-esterified fatty acids in the mice. Additionally, the expression of Sirt1 and p-AMPK*α*/AMPK*α* increased in both liver tissue and AML-12 cells, while lipid accumulation decreased [51]. These results indicate that rhein can effectively improve lipid metabolism in liver cells and restore liver function in NAFLD mice, potentially via the activation of the Sirt1/AMPK signaling pathway.

Furthermore, rhein can induce G1-phase arrest in rat mesangial cells (RMC) cultured under high-glucose conditions, upregulating pro-apoptosis mediators such as Bax and caspase-3. It also significantly reduces the area under the blood-glucose concentration curve and inhibits RMC proliferation [52]. The PI3K/Akt/FoxO1 signaling pathway is the main insulin signaling pathway regulating glucose metabolism [53]. Forkhead box protein O1 (FoxO1) is a multifunctional protein expressed in both pancreatic *β* cells and liver cells and is crucial for the maturation and function of *β* cells [54]. Akt is an important downstream molecule in the insulin signaling pathway, promoting glucose transport and glycogen synthesis and increasing insulin sensitivity. When Akt is activated, it phosphorylates FoxO1, rendering it inactive and then reducing gluconeogenesis and lowering blood glucose levels [55]. After rhein administration, studies observed increased activity of superoxide dismutase (SOD) and glutathione peroxidase (GSH-Px), alongside higher FoxO1 protein expression in type 2 diabetic rats. Blood sugar levels, kidney index, and MDA levels in renal tissue were decreased, as were the expressions of PI3K and Akt proteins [56]. Additionally, renal pathological damage improved, suggesting that rhein may protect the kidneys of type 2 diabetic rats by alleviating renal oxidative stress and regulating the PI3K/Akt/FoxO1 signaling transduction pathway. In conclusion, these findings suggest that rhein holds promise as a potential therapeutic agent for diabetes treatment.

Rhein has been shown to significantly increase energy consumption in obese mice, leading to weight loss, improved insulin resistance, and enhanced cognitive functions such as recognition and memory. This ultimately helps prevent obesity in mice fed a chronic high-fat diet [57]. In addition, rhein normalized serum levels of ALT and AST and reduced TG levels, fasting insulin concentration, and fasting plasma glucose in the liver, effectively inhibiting lipid accumulation and reversing steatosis [58]. Other studies demonstrated that, in db/db mice, rhein reduced the volume of white and brown adipocytes and decreased the levels of serum cholesterol and low-density lipoprotein (LDL). One potential mechanism for rhein’s reduction of fat content is its inhibition of PPAR*γ* activity and the expression of its target genes [59,60]. Additionally, rhein significantly reduced the expression of inflammation and oxidative damage markers, such as NF-*κ*B and 8-OHDG. It also improved glucose tolerance and protected pancreatic islet function in db/db mice [61].

Uncoupling protein 1 (UCP1) is the main effector molecule in brown fat, which is responsible for converting excess energy into heat. It is highly expressed in the inner membrane of brown fat mitochondria and is considered an important target for improving metabolic rate and reducing weight. Liver X receptors (LXRs), which play a crucial role in regulating cholesterol balance and lipid and energy metabolism, are also involved in this process. Rhein reduces the expression of LXR target genes, antagonizes the inhibitory effect of LXRs on UCP1, and increases the expression of UCP1 in adipose tissue, thus regulating metabolic disorders. This leads to a reduction in liver cell degeneration and improves liver function in obese mice with FLD caused by diet [62]. Moreover, rhein has been shown to reduce the body weight of rats, improve estrogen receptors (ERs) in both peripheral blood and adipose tissue, significantly lower PPAR*γ* levels, and upregulate insulin receptor (INSR) levels. These effects alleviate oxidative stress and pathological damage in the thoracic aorta caused by obesity [63]. Therefore, it is speculated that rhein may exert anti-obesity effects by regulating PPAR*γ*, INSR, oxidative stress, inflammation, and ERs.

### 3.2. Anti-Cancer Activity

Rhein and its derivatives have demonstrated promising therapeutic effects against several types of cancer, including liver, breast, lung, cervical, ovarian, and pancreatic cancer. Rhein controls the proliferation, apoptosis, invasion, and migration of cancer cells through multiple pathways, effectively inhibiting the occurrence and progression of various cancers (Table 1). Additionally, the _IC50_ values of rhein in various cancer cells are shown in Table 2.

#### 3.2.1. The Inhibitory Effect of Rhein on Liver Cancer

Liver cancer is one of the most common and aggressive malignant cancers worldwide. Studies have shown that rhein can significantly inhibit cell viability and induce apoptosis in liver cancer cells, such as Hep-G2, Huh7, HepaRG, and BEL-7402. Rhein achieves this by reducing cell colony formation, decreasing the proportion of G0/G1 phase cells, lowering the scratch healing rate, and decreasing the number of invading cells. It also downregulates the expression of c-Myc, cyclin D1, Ras and p-ERK/ERK protein while increasing the proportion of cells in the S phase and G2/M phase and upregulating p53 protein expression. Rhein inhibits the Ras/ERK signaling pathway, thus suppressing the proliferation, migration, and invasion in liver cancer cells. The _IC50_ value of rhein in HepG2 cells was found to be 1.615 × 10^5^ μmol/L at 24 h [64,65]. Rhein induces the production of reactive oxygen species (ROS), leading to mitochondrial membrane potential (MMP) loss, and arrest of the S phase of the cell cycle. This promotes apoptosis in HepG2 and Huh7 cells by inducing ROS production and activating the JNK/Jun/Caspase-3 signaling pathway [66]. The Caspase family plays an important role in apoptosis by disrupting DNA and inducing the formation of apoptotic bodies, primarily through Caspase-1, Caspase-3, and Caspase-9 [67]. In liver cancer cells, rhein has been shown to increase the protein expression of Fas, p53, p21, Caspase-3, Caspase-8, Caspase-9, and PARP. At the same time, it significantly reduces the protein levels of Bcl-2, cyclin A, CDK2, and oncogene c-Myc in B-lymphoblastoma, inducing the apoptosis of HepaRG [68] and BEL-7402 [69] cells by activating Fas and mitochondrial-mediated apoptosis pathways. The _IC50_ value for rhein in HepaRG cells at 24 h was 77.97 μmol/L. In summary, rhein shows considerable potential as a new therapeutic agent for liver cancer, and further research could lead to its development as a clinical treatment for this highly aggressive disease.

#### 3.2.2. The Inhibitory Effect of Rhein on Breast Cancer

Breast cancer is one of the most common and increasingly prevalent malignant cancers among women, making it a significant global public health concern [70]. Recent research has shown that NF-*κ*B plays a crucial role in cancer regulation [71]. Rhein has been found to inhibit HSP90*α* activity and promote the degradation of NF-*κ*B and COX-2 while also inhibiting the protein expression and phosphorylation level of HER-2. This in turn controls the proliferation and differentiation of breast cancer cells, particularly in SK-BR-3. The _IC50_ value of rhein for SK-BR-3 cells was 86 μmol/L [72,73]. These results indicate that rhein can promote the degradation of NF-*κ*B by inhibiting the PI3K/Akt/ERK pathway, thereby inhibiting tumor growth. Rhein has demonstrated significant inhibitory activity on two types of breast cancer cells (MCF-7 cells that overexpress HER7 (MCF-7/HER7) and control vector MCF-7 cells (MCF-7/VEC)). The _IC50_ values were 1.291 × 10^5^ μmol/L for MCF-7/VEC cells and 1.079 × 10^5^ μmol/L for MCF-7/HER2 cells [74]. In addition, rhein induced an S-phase arrest in MCF-7/HER7 cells and caused an increase in the G1 phase in MCF-1/VEC cells, thereby inhibiting cell proliferation. This effect is associated with the promotion of Caspase-9 expression. Rhein also induced apoptosis in MCF-7 cells through the paraptosis pathway, further contributing to its anti-breast cancer effects [75].

#### 3.2.3. The Inhibitory Effect of Rhein on Lung Cancer

Lung cancer is the first malignant tumor in China in terms of both incidence rate and mortality. The IL-6/STAT3 signaling pathway plays a crucial role in non-small cell lung cancer (NSCLC). Rhein has been shown to upregulate the expression of the pro-apoptotic protein Bax and downregulate the expression of the anti-apoptotic protein Bcl-2, inducing cell cycle arrest at the G2/M phase and promoting apoptosis in NSCLC cells. Rhein exerts its effects by mediating the IL-6/STAT3 signaling pathway, effectively controlling the occurrence and development of NSCLC. The _IC50_ values of rhein were 24.59 μmol/L in PC-9 cells, 52.88 μmol/L in H460 cells, and 23.9 μmol/L in A549 cells [76].

Cytochrome C (Cyt-C) is a soluble protein released into the cytoplasm by mitochondria in response to apoptosis stimuli and is a key factor in inducing cell apoptosis through the mitochondrial pathway, which induces cell apoptosis through the Caspase-dependent pathway [77,78]. Similarly, apoptosis-inducing factor (AIF) is a mitochondrial flavoprotein that also induces apoptosis. When apoptosis is triggered, mitochondrial membrane permeability increases, leading to the release of AIF into the cytoplasm and subsequent translocation to the nucleus, where it promotes DNA fragmentation [79,80]. Rhein can significantly increase the expression levels of Caspase-3, Caspase-9, Cyt-C, and AIF proteins in NSCLC A549 cells. It also induced G2-phase arrest and significantly reduced cell proliferation [81]. These findings suggest that rhein exerts its anti-NSCLC effects by regulating key protein expression pathways and inducing cell apoptosis.

#### 3.2.4. The Inhibitory Effect of Rhein on Other Cancers

In cervical cancer, rhein induces the degradation of *β*-Catenin and arrests the cell cycle in the S phase, thereby inhibiting the proliferation of Hela cells and inducing cell apoptosis in a dose-dependent manner [82]. In ovarian cancer cell lines (A2780 and OV2008), rhein reduces the production of ROS and inhibits the activity of NADPH oxidase and matrix metalloproteinases. This leads to a reduction in the expression and phosphorylation levels of JNK and AP-1 protein in the Rac1/ROS/MMPs signaling pathway, exhibiting the dose-dependent inhibition of cell proliferation and migration [83,84]. In pancreatic cancer, rhein significantly inhibits the proliferation of MiaPaCa-2 cells. Studies have shown that rhein reduces the expression of HIF-1*α* and vascular endothelial growth factor (VEGF) in pancreatic cancer cells under hypoxic conditions, and this inhibitory effect is dose- and time-dependent within a certain concentration range [85].

In colorectal cancer (CRC), rhein induces S-phase cell cycle arrest and increases the Bax/Bcl-2 ratio and the expression of activated Caspase-3 in SW480 cells. Rhein also directly targets and promotes mTOR degradation through the ubiquitin-proteasome pathway, thereby inhibiting the mTOR signaling pathway and reducing the expression of VEGF in CRC cells. This results in inhibited proliferation, invasion, migration, and induced apoptosis. The _IC50_ values of rhein in HCT15, HCT116, and DLD1 CRC cells at 24 h were 41.25 μmol/L, 47.77 μmol/L, and 46.51 μmol/L, respectively [86,87]. TRAIL, a member of the TNF superfamily, is widely expressed in various cells of the immune system and has been reported to inhibit tumorigenesis through NK cells and T cells [88]. In bladder cancer, rhein increases the expression of DR5 protein and DR5 mRNA at the transcriptional level in a dose-dependent manner. This effect reverses TRAIL resistance and enhances TRAIL-mediated apoptosis in bladder cancer cells without showing cytotoxicity to normal bladder epithelial cells [89]. In oral cancer, rhein inhibits the migration and invasion of oral cancer cells by regulating epithelial-mesenchymal transition (EMT)-related proteins. Additionally, rhein induces the accumulation of ROS and promotes apoptosis by inhibiting the AKT/mTOR signaling pathway, leading to significant inhibition of oral cancer cells [90].

**Table 1:** Anti-cancer effects of rhein in vitro.

Cancer Type	Cell Lines Used	Anti-Cancer Effect	Action Pathway	References
Liver cancer	HepG2 cells	Colony formation, G0/G1, scratch healing rate, cell invasion, c-Myc, cyclinD1, Ras, p-ERK/ERK↓S phase⊥G2/M, p53↑	Ras/ERK signaling pathway↓	[65,66]
	HepG2, Huh7 cells	ROS↑loss of MMP, S phase⊥	JNK/Jun/Case-3 signaling pathway↑	[67]
	HepaRG, BEL-7402 cells	Fas, p53, p21, Caspase-3/8/9, PARP↑ Bcl-2, cyclin A, CDK2, c-Myc↓	Apoptosis pathways↑	[69,70]
Breast cancer	SK-BR-3 cells	HSP90α↓NF-*κ*B, COX-2↓HER-2, PI3K, p-Akt, p-ERK↓	PI3K/Akt/ERK pathway↓	[73,74]
	MCF-7 cells	Caspase-9↑S phase⊥G7 phase↑	Para-apoptotic pathways↑	[75]
	4T1 cells	Caspase-3/8/9, Bax/Bcl-2↑	Exogenous apoptotic pathway↑	[76]
Lung cancer	PC-9, H460, A549 cells	Bax↑Bcl-2↓	IL-6/STAT3 signaling pathway↓	[77]
	A549 cells	Bcl-2↓ p-AMPK, LC3-II↑	PI3K/Akt/mTOR pathway↓	[82]
Cervical cancer	Hela cells	*β*-Catenin↓ S phase⊥	N/A	[83]
Ovarian cancer	A2780, OV2008 cells	ROS, JNK, AP-1, NADPH oxidase↓	Rac1/ROS/MMPs signaling pathway↓	[84,85]
Pancreatic cancer	MiaPaCa-2 cells	HIF-1*α*, VEGF↓	N/A	[86]
Colon cancer	SW480 cells	S phase⊥Bax/Bcl-2 ratio, Caspase-3↑ mTOR, VEGF↓	mTOR signaling pathway↓	[87,88]

Note: ↑, upregulate; ↓, downregulate; ⊥, arrest; N/A, not applicable.

**Table 2:** The _IC50_ values of the anti-cancer effect of rhein.

Cancer Type	Cell Lines Used	_IC50_ Value (μmol/L)	References
Liver cancer	HepG2 cells	1.615 × 10^5^	[65,66]
	HepaRG cells	77.97	[69]
Breast cancer	SK-BR-3	86.00	[73,74]
	MCF-7/VEC	1.291 × 10^5^	[75]
	MCF-7/HER2	1.079 × 10^5^	
Lung cancer	PC-9	24.59	[77]
	H460	52.88	
	A549	23.9	
Colorectal cancer	HCT15	41.25	[87,88]
	HCT116	47.77	
	DLD1	46.51	

#### 3.2.5. The Anti-Multidrug Resistance Effect of Rhein

Multidrug resistance (MDR) is a crucial challenge in cancer treatment and one of the important factors contributing to the failure of anti-cancer treatments in clinical practice [91]. Drug efflux transporters, especially P-glycoprotein (P-gp), play a major role in the development of MDR. Rhein has been shown to significantly downregulate the expression of P-gp through its interaction with STAT3, thereby inhibiting the efflux function of P-gp and achieving its effect as an MDR reverser [92]. By acting on the STAT3/Snail/MMP2/MMP9 pathway, rhein can significantly inhibit the proliferation and migration of chemosensitive and chemoresistant cancer cells [6]. Additionally, treatment with rhein increased the accumulation of doxorubicin (DOX) in the DOX-resistant cancer SMMC-7721 cell line, increasing the sensitivity of cancer cells to DOX while reducing their survival; the reverse effect may be achieved by inhibiting energy metabolism and inducing mPTP opening in cancer cells [93]. By improving the efficacy of anti-tumor drugs, rhein shows great potential in overcoming MDR in cancer treatment.

### 3.3. Anti-Inflammatory Effect

**Figure 3:**
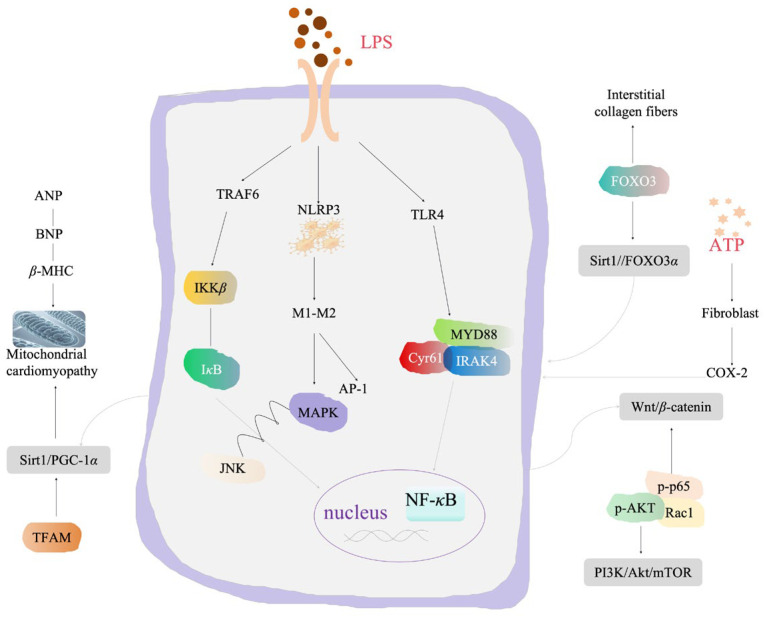
The pathway in anti-inflammatory effect of rhein. The black solid line represents the parallel relationship; the grey solid line arrow represents the indirect connection; the black solid line arrow represents the causal relationship; and the black wavy line represents the crosstalk relationship.

Rhein exhibits significant anti-inflammatory activity by inhibiting the activation of inflammatory cells, reducing the release of inflammatory factors, and inhibiting the activation of inflammatory signaling pathways, especially NF-*κ*B, thereby reducing the overall inflammatory response (Figure 3).

The PPAR*γ*/NF-*κ*B/HDAC3 signaling axis plays a key role in regulating inflammatory response. Studies have shown that rhein can promote the synthesis of a complex involving PPAR*γ*, NF-*κ*B, and HDAC3, which blocks the acetylation of NF-*κ*B and inhibits the release of downstream pro-inflammatory cytokines, thus reducing inflammation [94]. In vitro studies have found that rhein significantly reduces the expression of inflammatory factors, including TNF-*α, IL-1β*, IL-6, IL-12, and iNOS, while upregulating the expression of protective factor IL-10. Its anti-inflammatory mechanism is related to the regulation of multiple inflammatory signaling pathways such as PI3K/Akt, ERK1/2, and TLR4/NF-*κ*B [95]. Additionally, rhein has been found to lower the expression of proteins in the TLR2/NF-*κ*B signaling pathway, inhibiting the production and release of macrophage inflammatory factors induced by LPS [96]. In models of LPS-induced acute lung injury or acute respiratory distress syndrome, rhein significantly reduces the inflammatory reaction by targeting the NFATc1/Trem2 axis, which promotes the polarization of macrophages toward the anti-inflammatory M2 phenotype [97]. This suggests that rhein may be a promising candidate for the clinical treatment of inflammation-induced pathological injuries, particularly in the lungs. Given its efficacy in reducing pulmonary inflammation and damage induced by LPS, rhein could be clinically applied to various forms of lung injury.

Research has also shown that the abnormal activation of inflammatory pathways, particularly the TLR4/NF-*κ*B pathway, played a crucial role in the pathogenesis of NAFLD [98]. Rhein has been found to lower the expression levels of TLR4, MYD88, and Cyr61 in liver tissue, reduce lipid accumulation, and decrease the infiltration of inflammatory cells in liver cells [99]. These effects contribute to restoring the normal morphology and arrangement of nuclei, indicating a therapeutic role for rhein in treating diabetes complicated by NAFLD. In summary, rhein appears to improve liver function damage in NAFLD by inhibiting the TLR4 receptor pathway. In a study investigating the effects of rhein on experimental autoimmune encephalomyelitis (EAE) in mice, it was found that the down-regulation of IL-2 and the promotion of Foxp3 expression may be related to the effective mechanism of rhein in reducing the incidence [100]. Additionally, rhein has been shown to significantly reduce blood urea nitrogen, serum creatinine, and ROS levels while inhibiting the production of inflammatory molecules such as TNF-*α*, IL-6, and MCP-1 in LPS-induced models of kidney inflammation [101]. Rhein also weakens the activation of the NF-*κ*B pathway, further reducing inflammation. Furthermore, rhein can also activate the SIRT3/FOXO3*α* signal pathway, reduce renal interstitial collagen fibers, and significantly improve the morphology of damaged kidney tissue [102], this may explain the protective effects of rhein on kidney injury in SD rats undergoing 5/6 nephrectomy.

Diabetic cardiomyopathy (DCM) is one of the common causes of death among diabetes patients, and inflammation is a key factor in its pathogenesis. Rhein has been shown to reduce the production of inflammatory mediators such as NO, TNF-*α*, PGE2, and COX-2 in RAW264.7 cells stimulated by advanced glycation end products (AGEs), thereby alleviating cellular inflammatory damage. In vivo studies on DCM rats revealed that rhein inhibits the accumulation of AGEs and the infiltration of inflammatory factors in the heart while downregulating the expression of TRAF6 and inhibiting the phosphorylation of IKK*β* and I*κ*B. This suggests that rhein inhibits the NF-*κ*B pathway induced by AGEs/CM [103]. Interestingly, research has also found that JNK/MAPK can regulate the phosphorylation of I*κ*B without affecting IKK*β*, indicating that the anti-inflammatory mechanism of rhein may involve the inhibition of both the TRAF6-NF/*κ*B and JNK/MAPK pathways, with potential crosstalk between the two. Additionally, atrial natriuretic peptide (ANP) and B-type natriuretic peptide (BNP) are early markers of cardiac diastolic dysfunction, reflecting changes in atrioventricular pressure, and can be used for the early screening and prognosis of DCM [104]. The *β*-myosin heavy chain (*β*-MHC) is one of the myosin-heavy chains in the mammalian heart and is closely related to myocardial interstitial fibrosis, stiffness, and subsequent diastolic dysfunction, with its levels increasing in diabetic cardiomyopathy [105,106]. Research has confirmed that drugs targeting the Sirt1/PGC-1*α* pathway can effectively reduce myocardial damage [107,108], and it is speculated that rhein may treat DCM by regulating this pathway. In DCM mice, rhein significantly reduced blood glucose, ANP, BNP, and *β*-MHC mRNA and protein levels while reducing myocardial and mitochondrial damage. Additionally, rhein increased the expression of Sirt1, PGC-1*α*, and TFAM [109]. These findings suggest that rhein has a protective effect against myocardial injury in diabetes and could be a promising candidate drug for treating inflammation-related DCM.

Rhein has also shown a protective effect against intestinal damage, particularly in ulcerative colitis (UC). The PI3K/Akt/mTOR signaling pathway has been identified as an important target for UC treatment. Studies on rhein’s in vitro and in vivo anti-inflammatory effects in UC have demonstrated that rhein inhibits the production of pro-inflammatory cytokines, such as TNF-*α*, IL-6, IL-8, and IL-1*β*, while downregulating proteins related to the PI3K/Akt/mTOR signaling pathway [110]. Additionally, rhein reduces the expression of TLR4 and inhibits the phosphorylation of NF-*κ*B, alleviating intestinal inflammation induced by LPS via the TLR4/NF-*κ*B signaling pathway [111]. Rhein also regulates the TLR5/NF-*κ*B signaling pathway, reducing inflammatory cytokine secretion and lymphocyte infiltration, thereby alleviating symptoms such as colon shortening, weight loss, diarrhea, and bloody stools in UC rats [112]. The reduction of uric acid levels caused by rhein treatment is the key to improving colitis. By regulating the intestinal microbiota and reducing pathogenic bacteria, rhein indirectly alters gut purine metabolism, further alleviating the pathological symptoms of chronic colitis [113]. Rhein significantly reduces the secretion of IL-1*β* by interfering with the assembly of NLRP3 inflammasomes in macrophages and mediates the polarization of macrophages from M1 to M2 phenotype. It also interferes with the NF-*κ*B, AP-1, and MAPK signaling pathways, mitigating acute intestinal inflammatory response [114].

Rheumatoid arthritis (RA) is a chronic and systemic inflammatory disease that can lead to joint disability, although its exact pathogenesis remains unclear. Studies have confirmed that rhein can inhibit the release of tumor necrosis factor and pro-inflammatory cytokine interleukin in human osteoarthritis cartilage and synovial cells [115]. Rhein inhibits ATP-induced ROS production in synovial cells, reduces the expression of COX-2, IL-6, and MMP-9, and exhibits significant anti-inflammatory activity [116]. Rhein has also demonstrated efficacy in inhibiting the Wnt/*β*-Catenin signaling pathway in ectopic endometrial cells. By significantly reducing *β*-Catenin expression and its downstream targets, including p-p65, p-AKT, and Rac1, rhein inhibits the activation of NF-*κ*B and Wnt/*β*-Catenin signaling. This inhibition improves the proliferation and hypertrophy of the uterine myometrium, which may be beneficial in treating conditions such as endometriosis [117].

### 3.4. Anti-Fibrosis Effect

**Figure 4:**
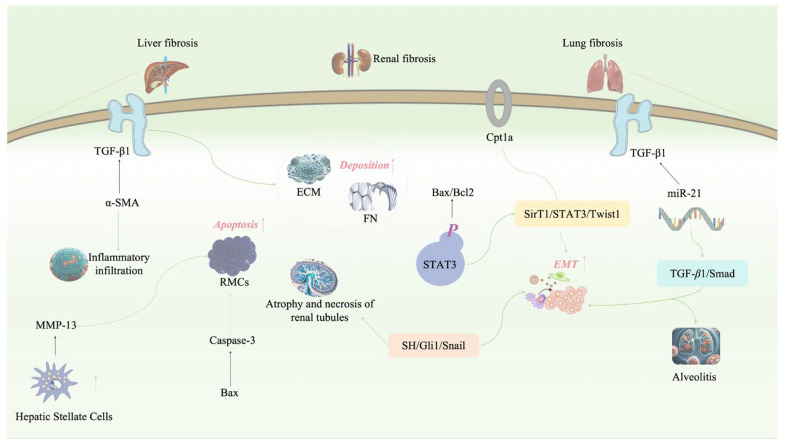
The pathway in anti-fibrotic effect of rhein. The black solid line arrow represents the parallel relationship, the green solid line arrow represents the promoting relationship, and the green dashed line arrow represents the inhibiting relationship.

Fibrosis is a pathological process that can occur in various organs and represents a repair response following tissue damage, which is intended to protect the structural integrity of tissues and organs. However, when this repair response becomes overly aggressive or uncontrolled, it leads to the excessive deposition of extracellular matrix (ECM) and a significant increase in fibrous connective tissue. This can ultimately disrupt normal tissue architecture, leading to organ dysfunction and, if left unchecked, chronic organ failure, a condition known as organ fibrosis [118]. Rhein has shown significant effects in improving fibrosis in various organs (Figure 4).

#### 3.4.1. Inhibition Effect on Liver Fibrosis

Numerous studies have shown that rhein has a strong inhibitory effect on liver fibrosis, and its ability to improve liver function may be primarily due to its anti-inflammatory properties. Transforming growth factor *β*1 (TGF-*β*1) plays a critical role in liver fibrosis, and its levels increase significantly during the development of the disease. By inhibiting the high expression of TGF-*β*1 and alpha-smooth muscle actin (*α*-SMA), rhein can significantly improve the structural disorder of liver lobules, reduce inflammatory infiltration, and alleviate liver fibrosis [119]. In addition, rhein inhibits the activation of hepatic stellate cells and upregulates the collagenase 3/*α*-SMA ratio, which promotes collagen degradation and reduces ECM secretion. This effectively inhibits both the occurrence and development of liver fibrosis [120].

#### 3.4.2. Inhibition Effect on Kidney Fibrosis

Kidney fibrosis is characterized by fibroblast activation and the excessive accumulation of ECM in the renal interstitium, a process that is involved in nearly all forms of chronic kidney disease. Around 40% of patients with IgA nephropathy (IgAN) will eventually progress to end-stage renal disease (ESRD), making IgAN one of the main causes of renal failure [121]. Glomerular sclerosis is the main pathological feature of ESRD, so preventing and treating this condition is critical in managing IgAN. Research has shown that rhein blocks the TGF-*β*1 pathway, reducing the expression of *α*-SMA and the deposition of fibronectin in renal interstitial fibroblasts, thereby inhibiting renal interstitial fibrosis and glomerular sclerosis. Rhein’s actions also help slow the progression of IgAN [122]. Diabetic nephropathy, another major cause of kidney fibrosis and a leading cause of death in diabetic patients, can also be improved by rhein [123]. Rhein acts via multiple pathways, including regulating glucose and lipid metabolism, reducing inflammatory responses, and alleviating oxidative stress [124,125]. In high-glucose conditions, rhein regulates the cell cycle in RMCs, upregulates apoptotic mediators such as Bax and Caspase-3, induces cell apoptosis, and inhibits RMC proliferation and ECM production, thereby improving renal fibrosis [52].

In unilateral ureteral obstruction (UUO) models, rhein reduces the expression of SHH, Gli1, and Snail, significantly improving renal interstitial fibrosis by regulating the SHH-Gli1-Snail signaling pathway. Rhein also reduces tubular atrophy and necrosis, interstitial fibrosis and proliferation, and abnormal ECM deposition [126]. Moreover, rhein exerts anti-fibrotic effects by inhibiting the expression of type I collagen and *α*-SMA after UUO. It also suppresses the phosphorylation of STAT3 and inhibits the expression of apoptosis-related proteins, including Bax and Bcl2 [127]. Partial epithelial-to-mesenchymal transition (pEMT) is considered one of the main causes of renal fibrosis, and fatty acid oxidation (FAO) dysfunction in renal tubular epithelial (RTE) cells plays a key role in this process. When carnitine palmitoyl transferase 1a (Cpt1a) activity is inhibited in RTE cells, it leads to ATP depletion and lipid deposition, which, in turn, induce EMT. Interestingly, rhein inhibits EMT, indicating that Cpt1a-mediated FAO dysfunction is crucial for EMT development in RTE cells. Further studies suggest that Cpt1a activity is regulated by the Sirt1/STAT3/Twist1 pathway [128]. Rhein promotes Cpt1a-mediated FAO via this pathway, thereby inhibiting EMT in RTE cells and improving renal fibrosis.

#### 3.4.3. Inhibition Effect on Lung Fibrosis

Rhein has been confirmed to have a significant inhibitory effect on pulmonary fibrosis. TGF-*β*1 is a key factor in promoting excessive ECM deposition, such as collagen, which contributes to the development of pulmonary fibrosis [129]. MiR-21 can upregulate the function of TGF-*β*1, further enhancing the fibrosis process. Rhein downregulates TGF-*β*1 expression by inhibiting miR-21, thereby intervening in the TGF-*β*1/Smad signaling pathway. This reduces ECM deposition, inhibits alveolitis, and decreases the degree of pulmonary fibrosis [10].

### 3.5. Cardiocerebral Protective Effect

**Figure 5:**
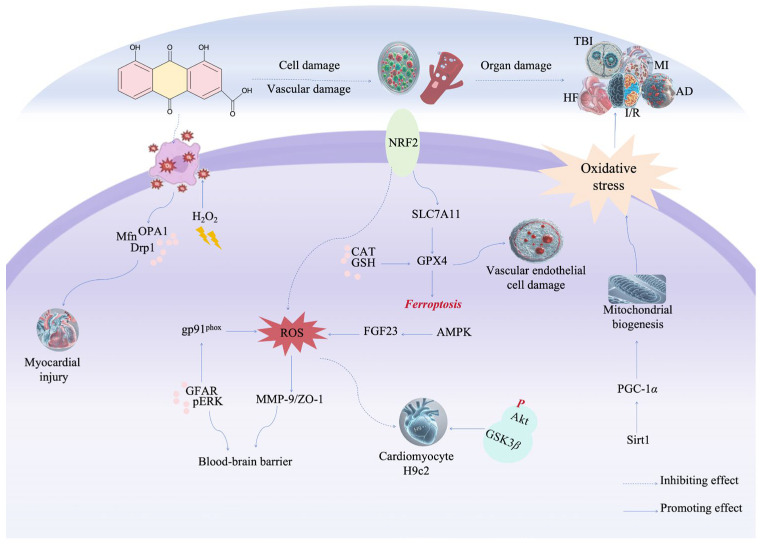
Cardiocerebral protective effect of rhein.

Rhein has shown a significant protective effect against vascular, cellular, and organ damage caused by oxidative stress (Figure 5). Rhein’s cardiocerebral protective properties stem from its ability to enhance antioxidant defenses by increasing the activity of SOD and catalase, significantly reducing the levels of oxidative markers like MDA and GSSG. It also boosts levels of NO, NOS, GSH, GSH-Px, and GSH/GSSG ratio, helping to repair endothelial cell damage caused by oxidative stress injury. Ultimately, these effects improve neurological function following cerebral ischemia-reperfusion (I/R) injury [130]. Its therapeutic effect on I/R brain injury both in vitro and in vivo is also mediated by the regulation of the PI3K/AKT/mTOR pathway, which reduces the levels of neuroinflammatory mediators including IL-6, IL-1*β*, and TNF-*α* [131]. In another study, Liu et al. examined the effects of rhein on I/R injury and ferritin deposition. They found that rhein inhibits oxidative stress and intracellular ROS production while increasing the protein expression of NRF2, SLC7A11, and GPX4 in both in vivo and in vitro models. Notably, the inhibition of ferroptosis was reversed when NRF2 was inhibited, confirming that rhein provides neuroprotection by regulating the NRF2/SLC7A11/GPX4 signaling pathway. This offers a potential therapeutic approach for treating ischemic stroke [132].

Oxidative stress can lead to blood–brain barrier (BBB) dysfunction after traumatic brain injury (TBI), mainly due to the upregulation of gp91phox, which regulates the macromolecular redox signaling pathway and significantly increases ROS level. ROS activates ERK1/2, which mediates the degradation of MMP-9 and ZO-1, leading to BBB dysfunction [133]. Rhein has been shown to downregulate the expression of MMP-9 mRNA and protein expression in control cortical impact rats while upregulating ZO-1 mRNA and protein expression. Additionally, rhein decreases the expression of GFAP and p-ERK, prevents gp91phox activation, and inhibits ROS production [134]. These findings suggest that rhein could serve as a potential therapeutic drug for restoring BBB function after TBI. Healthy mitochondria undergo continuous division and fusion, regulated by protein levels such as OPA1, Drp1, and Mfn [135]. Oxidative stress induced by H_2_O_2_ can disrupt the expression of these mitochondrial dynamic proteins and impair mitochondrial function [136]. Both rhein and its derivative, lysine rhein, have been shown to reverse the decrease in myocardial cell viability caused by H_2_O_2_, reduce myocardial cell apoptosis, and improve heart failure (HF) [137]. Rhein enhances the antioxidant capacity of rats by clearing free radicals and ROS, thereby protecting against acute cell damage [138].

Myocardial infarction is a serious cardiovascular disease that can lead to myocardial reperfusion injury, which promotes myocardial cell apoptosis and is closely related to HF [139,140]. Studies have shown that the Akt/GSK3*β* pathway plays a crucial role in cardiac protection [141]. Rhein significantly enhances the vitality of H9c2 cardiomyocytes and reduces cell apoptosis and production of ROS. In addition, rhein upregulates phosphorylation of Akt and GSK3*β*, regulating the Akt/GSK3*β* pathway to enhance the oxidative defense system and then participate in myocardial protection [142]. Rhein significantly inhibits the expression of FGF23 through the activation of AMPK, inhibits Ang II-induced cardiac hypertrophy and fibrosis through the AMPK/FGF23 axis, and improves cardiac systolic dysfunction. Furthermore, rhein significantly reduces the production of ROS and superoxide induced by Ang II [143].

Mitochondrial oxidative stress is a major factor in the pathogenesis of Alzheimer’s disease (AD). By regulating the Sirt1/PGC-1*α* pathway, rhein enhances mitochondrial biogenesis and increases SOD activity, reversing oxidative stress in the brain of AD mice. These antioxidant effects help alleviate cognitive impairment, suggesting that rhein could be a promising therapeutic agent for AD [144].

### 3.6. Antibacterial Effect

Rhein has been shown to possess strong inhibitory effects on both Gram-negative bacteria, such as *Escherichia coli* and *Helicobacter pylori*, and Gram-positive bacteria, including *Staphylococcus aureus*, *Bacillus subtilis*, *Bacillus anthracis*, and *Streptococcus* (Table 3). In addition, rhein demonstrates synergistic effects when combined with antibiotics [145].

*Staphylococcus aureus* is a widespread bacterium commonly found on human skin, especially in the nasopharynx [146]. Methicillin-resistant *Staphylococcus aureus* (MRSA) is characterized by its resistance to multiple drugs and the rapid evolution of this resistance, making MRSA one of the main pathogens in hospital infections [147] and an important challenge in clinical treatment. Rhein inhibits the growth and reproduction of MRSA by increasing the permeability of cell wall permeability, disrupting the integrity and stability of MRSA cell membranes, and inducing MRSA cell apoptosis. Similarly, in animal models, by disrupting bacterial biofilms, rhein inhibits the growth and reproduction of bacterial fluid [148], thereby reducing the bacterial load in the blood and major organs after MRSA infection [149].

*Streptococcus mutans* (*S. mutans*), the primary pathogenic factor for dental caries, is also susceptible to rhein. Rhein inhibits 90% of planktonic *S. mutans*, primarily by affecting the metabolism of biofilm cells and influencing biofilm formation, acidogenicity, and acid tolerance [150]. These findings suggest that rhein may offer a novel therapeutic approach for treating dental caries.

**Table 3:** Antibacterial and antiviral effects of rhein.

Disease Type	Pathogen	Pharmacodynamic Mechanism	References
N/A	MRSA	Permeability↑ and integrity and stability of MRSA cell membranes↓; MRSA⊥	[148]
Bacteremia	MRSA	Permeability of MRSA cell membranes↑; MRSA⊥	[149]
Dental caries	*S. mutans*	Biofilm formation⊥; Acidogenicity↓; Acid tolerance↓	[150]
Virus pneumonia	IAV	Oxidative stress↓; TLR4↓; Akt↓; p38↓; JNK/MAPK signaling pathway⊥; NF-*κ*B signaling pathway⊥	[15]
Hepatitis B	HBV	HBV reverse transcriptase↓; Synthesis of HBV DNA⊥	[151]

Note: ↑, upregulate; ↓, downregulate; ⊥, arrest; N/A, not applicable.

### 3.7. Antiviral Effect

Rhein has demonstrated notable antiviral activity (Table 3), particularly against the influenza A virus (IAV), a highly pathogenic virus that has caused multiple global pandemics and is prone to rapid mutation. Wang et al. found that rhein enhances the expression of TLR4, Akt, p38 protein, and c-Jun amino-terminal kinase while also activating the NF-*κ*B signaling pathway. These actions reduce oxidative stress induced by IAV and inhibit adsorption, replication, and diffusion. In vivo studies on IAV-infected mice showed that rhein reduces the production of inflammatory factors in the lungs, improves lung tissue pathology, and increases survival rates [15]. Additionally, rhein has been proven to be a reversible non-competitive reverse transcriptase inhibitor. By combining with hepatitis B virus (HBV) reverse transcriptase, rhein makes the enzyme unable to produce activity, thus inhibiting the synthesis of HBV DNA and treating hepatitis B [151].

### 3.8. Other Pharmacological Effects

The CDK9 gene encodes a protein that belongs to the cyclin-dependent kinase (CDK) family and plays a key role in regulating the cell cycle. Rhein has been confirmed to inhibit the expression and phosphorylation of CDK9 and STAT3, leading to improvements in pulmonary vascular remodeling and right myocardial hypertrophy. These effects suggest that rhein could be a potential therapeutic agent for treating hypoxic pulmonary hypertension in rats [152]. Rhein can relax airway smooth muscle by inhibiting L-type voltage-dependent Ca^2+^ channels on the cell membrane, preventing Ca^2+^ influx and thus reducing airway constriction [153]. Rhein exhibits antiplatelet aggregation effects by acting as a P2Y12 receptor antagonist. The P2Y12 receptor is commonly targeted by antiplatelet drugs, and studies suggest that rhein and its derivatives have a high affinity for the P2Y12 receptor, displaying properties similar to conventional P2Y12 antagonists [154]. This makes rhein a promising candidate for preventing platelet aggregation. Endometriosis, a common gynecological condition, involves the proliferation, migration, and invasion of endometrial stromal cells. Rhein has been shown to inhibit these processes by targeting micro RNA-135, exerting anti-proliferative effects, and potentially offering a new treatment avenue for endometriosis [155].

### 3.9. Clinical Application

As a plant-derived active natural product, rhein has been applied in modern clinical practice. Chronic constipation is a common disease, especially in middle-aged and elderly patients, seriously reducing their quality of life [156]. Western medicinal laxatives are easily accessible; however, their use is often associated with side effects, limiting their application among middle-aged and elderly populations [157]. Recent findings suggest that butyrate production by the gut microbiota effectively regulates constipation in middle-aged individuals [158,159]. After supplementing with rhein in middle-aged patients with constipation, an increase in the abundance of *Lachnospiraceae*, including *Agathobacter* and *Roseburia*, was observed in the composition of the gut microbiota, which subsequently regulated butyrate and stool consistency [160]. These changes alleviate chronic constipation in middle-aged patients. Rhein was detected in the urine and serum samples of patients with epidermolysis bullosa simplex (EBS) treated with diacerein for four weeks. As an active metabolite of diacerein absorbed through the skin, rhein can exert long-lasting anti-inflammatory activity. The oral administration of rhein significantly reduces blister formation in EBS patients without causing side effects or complications, offering promising therapeutic strategies for EBS treatment [161].

## 4. Pharmacokinetics

The pharmacokinetic characteristics of rhein differ across species [162,163], and current pharmacokinetic studies have been conducted in rats, mice, rabbits, and beagles (Table 4). These studies indicate that after oral administration, rhein is most abundantly distributed in liver tissue, followed by the stomach, intestine, lung, spleen, kidney, heart, and brain. Rhein absorbed into the body is first metabolized into its glucuronide and sulfate forms, which are then excreted through bile into the hepatic-intestinal circulation. In the intestine, glucuronide and sulfate groups are removed, allowing it to be reabsorbed or converted into rhein anthraquinone compounds.

When five classic anthraquinone glycoside monomers from rhubarb were administered simultaneously, the average drug-time curves for the five glycoside monomers showed varying degrees of double peaks, despite all sharing the basic anthraquinone parent nucleus structure. Among them, rhein demonstrated significantly higher AUC _(0-t)_, AUC _(0-∞)_, and C_max_ values compared to emodin, aloe emodin, emodin methyl ether, and emodin phenol. However, there were no significant differences in half-life, clearance rate, and dosage across varying doses of rhein, suggesting that rhein exhibits linear pharmacokinetic properties.

Rhein is mainly administered orally, and pharmacokinetic studies have confirmed that it is rapidly absorbed and has a short half-life following oral administration. This rapid absorption underscores the importance of selecting the optimal medication time for clinical application. Additionally, the detection of rhein in brain tissue indicates that it can cross the BBB, suggesting potential use in the treatment of brain diseases.

## 5. Toxicology

In recent years, with the increasing global recognition of China and TCM, TCM has gained more attention internationally. However, concerns about its potential toxic effects have also emerged, which have constrained its development in the international medical field. Therefore, evaluating the safety and toxicity of TCM and its active ingredients is crucial.

The liver plays a key role in drug metabolism, as any drug that enters systemic circulation is frequently metabolized by the liver. Similarly, the kidneys maintain the homeostasis of the body, regulate metabolites, and balance acid-base levels, making them one of the most important organs in the human body. Consequently, liver toxicity and nephrotoxicity are primary concerns in the evaluation of TCM toxicity. Many TCM formulations that contain rheum anthraquinone compounds have been reported to cause liver and kidney toxicity, and these compounds often have a relatively high rhein content [170]. Interestingly, as discussed in the pharmacology section, rhein has shown protective effects on the liver and kidneys by inhibiting TGF-*β*1 to improve fibrosis, promoting uric acid excretion, and regulating the NF-*κ*B pathway. However, studies have also shown that rhein exerts a protective effect that is dose-dependent; at doses below 100 mg/kg with short-term administration, rhein exhibits protective effects, while the oral administration of rhein exceeding 175 mg/kg for 60 days can lead to severe nephrotoxicity. This suggests that the hepatoprotective and renal protective effects of rhein, alongside its potential toxicity, are mainly related to the concentration and duration of administration.

### 5.1. Hepatotoxicity

In a 75-day subchronic toxicity study, elderly mice treated with a daily dose of 375 mg/kg of rhein exhibited a 55.5% mortality rate, while no deaths were observed in young mice. The acute toxicity test study found that the LD50 of rhein was 2185.6 mg/kg. Additionally, typical pathological injuries in liver tissue were observed in the elderly group. It can be inferred that rhein-induced oxidative stress, mitochondrial dysfunction, and activated apoptosis lead to liver tissue damage [171].

Rhein significantly reduces the activity of CYP2C19, the main metabolic enzyme responsible for rhein metabolism. It activates rhein to generate active metabolite epoxides, which bind to intracellular mitochondria subsequently, leading to the excessive production of ROS and an increased AST level. In a primary rat liver cell model with low CYP450 enzyme activity, rhein was found to damage the liver and disrupt the mitochondrial respiratory chain, leading to cell death. However, the administration of CYP2C19 inhibitors restored mitochondrial membrane potential and AST levels, further indicating that CYP2C19 mediates the hepatotoxicity of rhein [172].

Another enzyme involved in rhein’s hepatotoxicity is UDP glucuronosyl transferase 1A1 (UGT1A1), an important phase II metabolic enzyme. UGT1A1 metabolizes drugs and produces glucoside and glucuronic acid compounds for excretion through bile. It is also the only enzyme that metabolizes the endogenous substance bilirubin. The phase *I* metabolite of rhein, rhein hydroxylate, significantly reduces the activity of the UGT1A1 enzyme, leading to abnormal drug and bilirubin accumulation and subsequent hepatotoxicity in the body, causing the accumulation of bilirubin and resulting in hepatotoxicity [173].

### 5.2. Nephrotoxicity

The long-term high-dose administration of rhein has been associated with nephrotoxicity in mice. After 60 days of treatment with rhein, both blood urea nitrogen and serum creatinine levels were elevated in the rhein-treated mice. After 30 days, mice in the high-dose group showed significant reductions in body weight and SOD activity and significant increases in TNF-*α* and expression of Caspase-3. In male mice receiving high doses of rhein, the expression of TGF-*β*1 was enhanced, while GSH-Px levels and the renal index were significantly decreased, and pathological changes were observed in kidney tissue morphology [174]. These results suggest that long-term, high-dose oral administration of rhein can lead to nephrotoxicity in mice. The potential toxic mechanisms of rhein’s nephrotoxicity include excessive oxidative stress, triggering inflammatory reactions, and promoting cell apoptosis.

## 6. Derivatives of Rhein

Rhein suffers from poor solubility and low bioavailability, which severely limit its clinical applications. To address these issues, researchers have modified the structure of rhein to create compounds with enhanced activity and greater potential for clinical development. One of the most representative derivatives is diacerein, a diacetate product formed by modifying the hydroxyl group of rhein. Diacerein has been widely used in clinical practice to treat osteoarthritis [175]. Encouraged by these advances, numerous researchers have focused on the structural modification of rhein and achieved promising results. Additionally, coupling rhein with other active ingredients can also enhance its efficacy, reduce side effects, and improve drug selectivity through synergistic effects [176].

### 6.1. Structural Modifications of Rhein

**Figure 6:**
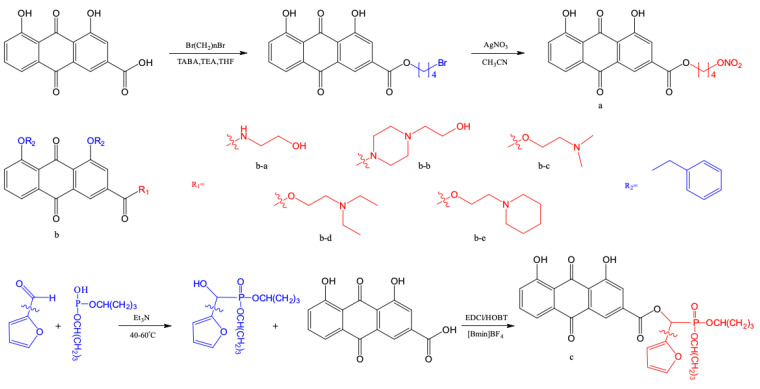
Derivatives of rhein. The blue part represents the substrates for synthesizing rhein derivatives or the substituents they provide; The red part represents the new functional groups introduced into the structure of rhein.

A series of rhein derivatives have been synthesized by introducing NO donor groups through alkyl chains of different lengths at the 2-carboxyl group of rhein. These derivatives exhibited stronger inhibitory activity on HepG2 cell proliferation than rhein itself. Structure–activity relationship studies showed that compound a (Figure 6), synthesized with a 3-carbon alkyl chain as the connecting arm, showed better cytotoxicity against HepG2 cells than the positive control drug 5-Fluorouracil [177].

In another study, a nucleophilic substitution reaction was performed using benzyl chloride with rhein as the starting material, followed by hydrolysis, acylation, condensation, and other reactions to synthesize a new compound b (Figure 6). These compounds exhibited significant anti-proliferative effects on human nasopharyngeal carcinoma cells (CNE-1 and CNE-2), and human liver cancer cells (SMMC-7721 and HepG2) [178]. Notably, the derivative b-a showed a particularly strong inhibitory effect on the proliferation of ovarian cancer cells (SKOV3, SKOV3-PM4, and A2780) [179]. Additionally, b-b downregulated the expression of rac1 protein in breast cancer cells, leading to microfilament rearrangement and inhibition of cancer cell invasion and metastasis, without being toxic to normal human breast cells [180]. These findings indicate that the type of substituent on the 2-position carboxyl group has a significant impact on the anticancer activity of the target compound. Further studies revealed that compound b-a exerts a stronger tumor-killing effect than rhein at the same concentration. It is speculated that the introduction of a diphenyloxy group at the 1,8-positions of the anthraquinone ring plays an important role in sustaining the effects, and this activity may be related to the presence of a substituent at the C-3 position of the anthraquinone ring.

Using the fragment-based drug discovery method, Yang et al. synthesized a derivative compound c (Figure 6) by introducing *α*-hydroxyphosphate into the 2-carboxyl group of rhein [181]. This derivative exhibited significantly better inhibitory activity against MRSA than rhein and the positive control drug benzylpenicillin. These findings provide experimental and theoretical support for the development of new anti-drug-resistant bacteria drugs. The structural optimization and antibacterial mechanism of these derivatives deserve further investigation to identify potential candidate compounds against MRSA.

Introducing amino and nitrate groups at the 2-carboxy position of rhein enhances the inhibitory effect of the derivatives on various tumor cells, including lung cancer and liver cancer, surpassing the activity of the parent compound. This suggests that structural modifications of rhein may be an effective strategy for designing new anti-tumor drugs and developing potential treatments for cancer and chemotherapy. Additionally, introducing piperazine fragments into rhein derivatives can enhance their binding affinity to target proteins, while adding phosphate groups can improve antibacterial activity. These structural modifications provide new insights for further research on improving the physicochemical properties and biological activities of rhein derivatives.

### 6.2. Twin Drugs of Rhein

Twin drugs are new molecules formed by covalently linking two identical or different lead compounds or drugs. This approach aims to enhance pharmacological activity, generate new therapeutic effects, or improve drug selectivity. Twin drugs are designed with the expectation of achieving high therapeutic efficacy while minimizing adverse reactions, and many twin drugs have already been successfully applied in clinical practice.

#### 6.2.1. Derivative of Rhein and Taurine

As previously mentioned, rhein can reduce serum uric acid and improve kidney injury. Similarly, taurine lowers xanthine oxidase (XOD) activity, thereby reducing uric acid levels and improving kidney function. Given their similar pharmacological effects, researchers have combined rhein with taurine to create a rhein–taurine complex. This complex is synthesized by reacting rhein with dichlorosulfoxide to generate rhein acyl chloride, which is then connected to taurine through amide bonds. The resulting compound shows significantly stronger inhibitory activity on XOD than either rhein or taurine alone. It reduces uric acid production, promotes its excretion through the intestines, and demonstrates concentration-dependent inhibition [182].

#### 6.2.2. Derivatives of Rhein and Diacerein

Mycobacterium tuberculosis, the pathogen responsible for tuberculosis, is the ninth leading cause of death globally. The development of drug resistance has strengthened an urgent need for new anti-tuberculosis agents. While rhein is a natural product with antibacterial activity, its poor lipid solubility prevents it from penetrating the cell wall and thus limits its activity against tuberculosis. By chemically modifying rhein through its reaction with diacerein, a more lipophilic structure can be obtained, enabling the compound to enter cells and exhibit significant inhibitory activity against mycobacterium tuberculosis. Additionally, some of these derivatives exhibit reduced toxicity [183].

#### 6.2.3. Derivatives of Rhein and Niacin

Thrombotic diseases have high incidence and mortality rates, making them a serious threat to human health. Anti-platelet drugs are commonly used to reduce the risk of thrombosis. Rhein has been shown to inhibit platelet aggregation, which helps prevent thrombus. Niacin is effective at lowering blood lipids, dilating peripheral blood vessels, and inhibiting platelet aggregation. Researchers have synthesized a rhein–niacin conjugate by coupling rhein and niacin through alkane chains. This conjugate demonstrates anti-platelet aggregation activity that surpasses that of the positive control drug aspirin [184].

#### 6.2.4. Derivatives of Rhein and Paeonol

Osteoarthritis is a chronic degenerative disease commonly affecting middle-aged and elderly populations, leading to a significant reduction in the quality of life of patients worldwide [185]. As discussed earlier, rhein possesses strong anti-inflammatory activity. Paeonol, the main active component in the TCM Mudan Pi, also exhibits anti-inflammatory, anti-platelet aggregation, and other biological activities, and is mainly used for antipyretic, analgesic, and anti-inflammatory treatments in clinical practice [186]. Using the principle of molecular assembly, rhein and paeonol have been coupled with alkane chains of varying carbon lengths to create rhein–paeonol conjugates. These conjugates inhibit the expression of inflammatory factors induced by LPS and show improved solubility in organic solvents such as ethanol compared to rhein alone [187].

#### 6.2.5. Derivative of Rhein and Cisplatin

A novel compound has been synthesized by connecting cisplatin to rhein through ester bonds after oxidation. This compound effectively enters human lung cancer A549/DDP cells and induces mitochondrial damage. Its cytotoxicity is higher than that of cisplatin in lung cancer cells, but notably lower than cisplatin in normal human liver cells (HL-77020), indicating enhanced safety. This derivative is expected to be a potential mitochondrial-targeted drug for lung cancer treatment [188].

#### 6.2.6. Derivatives of Rhein and Methotrexate

A targeted rhein-methotrexate (RH-MTX-SLN) formulation in the form of solid lipid nanoparticles (SLNs) was developed for the treatment of RA. When applied to animal models of adjuvant arthritis induced by ERs, RH-MTX-SLNs significantly reduced inflammation and arthritis markers, improved ERS-mediated apoptosis, and demonstrated therapeutic efficacy in treating RA [189].

By combining rhein with other compounds that share similar pharmacological activities, researchers have been able to create derivatives that reduce toxicity and increase efficacy, much like the compatibility of TCM formulas. These findings suggest that further exploration of new methods for modifying the chemical structure of rhein could yield more optimized options for its clinical use.

## 7. Combination Therapy of Rhein

Recent medical research has shown that many diseases have complex pathogenesis, which can make traditional drug treatments less effective. In recent years, the combination of two or more drugs has shown significant advantages in the clinical management of various conditions, including cancer, cardiovascular disease, and bacterial infections, often leading to greater therapeutic effects compared to a single drug alone [190].

Rhein has demonstrated a synergistic effect when combined with oxaliplatin in the treatment of pancreatic cancer. By increasing the production of intracellular ROS and inhibiting the activation of the PI3K/AKT signaling pathway, the combination promotes the apoptosis of pancreatic cancer cells. Compared with single-drug administration, rhein enhances the sensitivity of pancreatic cancer cells to oxaliplatin, leading to a stronger anti-tumor effect. This indicates that the combination of rhein and oxaliplatin could be an effective strategy for overcoming drug resistance in the treatment of pancreatic cancer [191]. Similarly, studies have confirmed that the combination of rhein and paclitaxel (PTX) has a significantly synergistic effect against NSCLC. In comparison to PTX alone, the combined treatment simultaneously induces greater apoptosis and significantly increases the toxicity of PTX in NSCLC A549 cells in a concentration-dependent manner. This enhances PTX-induced autophagy in A549 cells by inhibiting the PI3K/AKT/mTOR pathway to enhance PTX-mediated autophagy and influencing Bcl-2 family proteins, thereby affecting cell apoptosis [192]. Western blot analysis demonstrated that rhein enhances the anti-tumor activity of PTX by affecting key proteins involved in apoptosis, such as Caspase-3 and PARP cleavage fragment proteins [193].

Atezolizumab is often used to treat bladder cancer, lung cancer, and breast cancer that are resistant to chemotherapy. In studies involving 4T1 breast cancer xenograft mice, rhein, when used alone or in combination with atezolizumab, significantly increased the mRNA levels of Caspase-3, Caspase-8, Caspase-9 and Bax/Bcl-2 in tumor tissues, promoting cell apoptosis. While all treatment groups showed tumor growth inhibition, the combination treatment exhibited the most pronounced effect, indicating a potential synergistic relationship between rhein and atezolizumab in cancer therapy [194].

*C. trachomatis*, a gram-negative bacterial pathogen, is a common sexually transmitted infection that can lead to serious complications [195]. Azithromycin is widely used to treat trachoma infections, but treatment failures due to antibiotic resistance are common [196,197]. Rhein exerts a significant inhibitory effect on *C. trachomatis* by regulating host cells and altering the bacterial environment. When combined with azithromycin, the two drugs synergistically enhance their inhibitory effects both in vitro and in vivo [198], indicating that rhein may play a role in developing new treatments for *C. trachomatis*. In another study, rhein was combined with metronidazole or other polyphenols to combat the periodontal pathogen *Porphyromonas gingivalis*. Rhein significantly reduced the expression of genes, such as fimA, hagA, and hagB, which are involved in host colonization, and downregulated protease genes like rgpA and kgp related to immune defense mechanism inactivation, tissue destruction, and nutrient acquisition, weakening the pathogenic effect of periodontal disease pathogen *Porphyromonas gingivalis* [199].

Curcumin, derived from the dried rhizomes of turmeric, shares similar biological activities with rhein. When combined, curcumin and rhein demonstrate significantly enhanced effects on reducing renal injury, collagen deposition, and inflammatory cell infiltration at corresponding time points, which can effectively treat renal interstitial fibrosis [167]. This combined effect may stem from the inhibition of metabolic enzymes, which increases the in vivo distribution of the drugs, leading to more effective treatment outcomes. Catalpol, another compound with anti-inflammatory properties, has been combined with rhein to treat EAE in mice. This combination reduced the infiltration of pro-inflammatory T cells into pathological lesions while increasing the levels of anti-inflammatory factors such as GATA3, Foxp3, IL-4, and IL-10. At the same time, it significantly reduced the expression of pro-inflammatory factors such as T-bet, ROR-*γ*T, IL-2, and IL-17 to regulate the inflammatory response and immune function [200].

## 8. Discussion

### 8.1. Current Status and Limitations

Plant-derived natural compounds have become a key focus in contemporary medical research, serving as valuable sources for developing new drugs to treat a variety of diseases. Rhein, a compound widely distributed in TCM, is relatively easy to obtain and has garnered increasing attention for its comprehensive pharmacological activities. Recent research both in vitro and in vivo has convincingly demonstrated that rhein has potential in hepatic and renal protection, anti-cancer, anti-inflammatory, antioxidant, and antibacterial activities. These findings suggest that rhein has significant therapeutic potential in treating liver and kidney diseases, cancers, inflammatory diseases, bacterial infections, viral infections, and other conditions.

Although rhein has demonstrated a wide range of pharmacological activities, it also has potential toxic side effects, particularly concerning the liver and kidneys. Rhein exhibits both protective and toxic effects on these organs, which appear to be closely related to the dosage and duration of administration. Therefore, the administration dosage and the length of the treatment cycle are crucial in maximizing rhein’s therapeutic benefits while minimizing toxicity. The current incomplete toxicological research on rhein makes it difficult to ensure the safety of its clinical use. In addition, pharmacokinetic studies indicate that rhein is well absorbed and quickly distributed to various organs, followed by rapid elimination. However, current studies do not provide a complete understanding of rhein’s absorption, distribution, metabolism, and excretion processes.

Rhein exhibits strong polarity, low solubility, poor oral bioavailability, and has been prone to cause gastrointestinal diseases [201,202], which limits its clinical application. Fortunately, the improvement in extraction and separation levels and the maturity of synthesis technology have provided convenience for the research of rhein. Numerous rhein derivatives have emerged, which exhibit improved solubility and bioavailability, expanding their pharmacological range while reducing toxicity. These structural modifications, for example, have enhanced rhein’s anti-cancer effects and mitigated some of its side effects. Furthermore, the implementation of the twin drug strategy has achieved effects such as enhancing drug efficacy, improving drug resistance, and increasing solubility, and the application of twin drugs including nanoformulation technology has even achieved targeted drug delivery.

### 8.2. Recommendations for Future Studies

To address current challenges and further the development of rhein, we propose the following suggestions.

Firstly, more comprehensive studies are needed to elucidate the molecular mechanisms underlying rhein’s biological activities. Future research should focus on using diverse cells and animal models to explore the comprehensive regulatory effects of rhein on signaling pathways, cell proliferation, and apoptosis during disease progression and treatment. Metabolomics, transcriptomics, and proteomics can identify differentially expressed metabolites, genes, and proteins, enabling a comprehensive elucidation of rhein’s molecular mechanisms and pharmacological targets.

Secondly, current research on rhein’s pharmacokinetics is limited. Future studies should investigate the pharmacokinetic characteristics of rhein across different administration methods and animal species to provide a solid scientific foundation for clinical application. In addition, more comprehensive and systematic toxicological research on rhein is required to confirm its toxicity and mechanisms, ensuring its safe and effective use in clinical trials.

Finally, to improve the physical and chemical properties of rhein and enhance its pharmacological activity, it is necessary to focus on developing structural modifications of rhein. These modifications could yield compounds with higher activity, improved safety, and better targeting. Existing research has modified the structures of rhein’s carboxyl group at position 2, hydroxyl groups at positions 4 and 5, and the atomic nucleus at positions 6 and 7. Moving forward, continued efforts to diversify and optimize rhein derivatives may open new avenues for their application in clinical settings. In addition, nanopreparations offer a promising solution with drug formulations featuring nanoscale properties that can compensate for the low solubility and gastrointestinal damage of rhein. Twin drug preparations can increase the solubility of rhein, enhance its pharmacological effects, improve drug resistance, and achieve targeted drug delivery, which is also an area that should be emphasized in development. Future research should integrate pharmacology, medicinal chemistry, and computer-aided drug design, leveraging multiple technologies to facilitate the design and synthesis of safer and more effective new derivatives. Moreover, as a key ingredient in TCM, rhein holds promise for combination therapy, providing potential benefits for optimizing clinical treatment strategies and developing new drugs. These developments are expected to successfully bring rhein to the market.

## 9. Conclusions

Rhein exhibits a wide range of pharmacological activities and has minimal side effects, making it a highly promising plant-derived natural compound. This review comprehensively summarizes the key aspects of rhein, including its TCM sources, extraction and separation techniques, pharmacological activities and mechanisms, pharmacokinetic distribution, toxicology, structural modifications, and combination therapy studies, highlighting its potential challenges and future prospects. We aim for this review to offer perspectives for future research on rhein and serve as a valuable reference for its further development and rational application in clinical trials.

## Figures and Tables

**Table 4:** Pharmacokinetics of rhein.

Species	Substance	Route	Dosage (mg/kg)	Pharmacokinetic Parameters							Reference
	of Administration			AUC_(0–t)_ (μg/mL·h)	C_max_ (μg/mL)	t_max_(h)	t_1/2_(h)	MRT(h)	CL (L/h· kg)	Vd(L/kg)	
KM mice	Rhein	i.g.	34.50	13.41 ± 6.64	2.98 ± 1.38	0.51 ± 0.12	11.71 ± 5.42	7.19 ± 2.69	8.29 ± 17.45	50.99 ± 50.55	[164]
			17.25	3.70 ± 1.02	1.55 ± 0.43	0.46 ± 0.17	9.19 ± 3.35	6.36 ± 2.16	3.88 ± 1.46	53.13 ± 25.14	
KM mice	Rhein	i.g.	34.50	8.04 ± 2.72	2.31 ± 0.92	0.49 ± 0.15	9.03 ± 3.78	7.79 ± 3.05	3.60 ± 1.13	46.85 ± 21.57	[165]
			69.00	16.42 ± 6.71	3.95 ± 1.25	0.56 ± 0.19	9.81 ± 4.25	7.53 ± 2.57	3.43 ± 1.02	48.54 ± 17.38	
SD rats	ZZDH Tang	i.g.	N/A	668.20 ± 170.20	357.20 ± 90.60	0.22 ± 0.08	3.42 ± 0.67	2.90 ± 0.67	3.80 ± 1.10	N/A	[166]
SD rats	Rhein	i.g.	100.00	18.96 ± 2.26	4.82 ± 0.70	0.49 ± 0.15	6.90 ± 5.40	N/A	4.91 ± 0.94	44.20 ± 27.20	[167]
Rabbits	SH Powder	Ap.	N/A	2.91 ± 0.88	0.48 ± 0.09	24.66 ± 7.03	10.28 ± 4.07	29.44 ± 6.56	0.0013 ± 0.0002	0.018 ± 0.013	[168]
Beagles	Rhein	i.v.	0.40				*α*: 0.05 ± 0.07				[169]
				1.50 ± 0.49	3.81 ± 0.65	N/A	*β*: 0.44 ± 0.52	0.95 ± 0.22	0.29 ± 0.10	0.72 ± 0.33	
							*γ*: 1.77 ± 0.93				
			20.00				ka: 0.52 ± 0.52				
				41.02 ± 17.64	9.71 ± 4.89	2.75 ± 1.84	*α*: 1.88 ± 0.43	4.29 ± 0.57	0.56 ± 0.21	2.72 ± 1.29	
							*β*: 3.25 ± 0.85				

Note: AUC_(0–t)_, area under the concentration-time curve from 0 to t hours; C_max_, maximum plasma concentration; tmax, time to maximum plasma concentration; t_1/2_, elimination half-life; MRT, mean residence time; CL, clearance; Vd, apparent volume of distribution; i.g., intragastric administration; i.v., intravenous administration; Ap., Application; N/A, not applicable; ZZDH Tang, Zhizi Dahuang Tang; SH Powder, Shenhuang Powder.
